# Comparative study of thermally activated delayed fluorescent properties of donor–acceptor and donor–acceptor–donor architectures based on phenoxazine and dibenzo[*a,j*]phenazine

**DOI:** 10.3762/bjoc.18.48

**Published:** 2022-04-25

**Authors:** Saika Izumi, Prasannamani Govindharaj, Anna Drewniak, Paola Zimmermann Crocomo, Satoshi Minakata, Leonardo Evaristo de Sousa, Piotr de Silva, Przemyslaw Data, Youhei Takeda

**Affiliations:** 1 Department of Applied Chemistry, Graduate School of Engineering, Osaka University, Yamadaoka 2-1, Suita, Osaka 5650871, Japan; 2 Faculty of Chemistry, Silesian University of Technology, Strzody 9, 44-100 Gliwice, Poland; 3 Department of Energy Conversion and Storage, Technical University of Denmark, Anker Engelunds Vej 301, 2800 Kongens Lyngby, Denmark

**Keywords:** charge-transfer, dibenzophenazine, donor–acceptor, organic light-emitting diodes, thermally activated delayed fluorescence

## Abstract

A new thermally activated delayed fluorescence (TADF) compound based on a donor–acceptor (D–A) architecture (D = phenoxazine; A = dibenzo[*a,j*]phenazine) has been developed, and its photophysical properties were characterized. The D–A compound is applicable as an emitting material for efficient organic light-emitting diodes (OLEDs), and its external quantum efficiency (EQE) exceeds the theoretical maximum of those with prompt fluorescent emitters. Most importantly, comparative study of the D–A molecule and its D–A–D counterpart from the viewpoints of the experiments and theoretical calculations revealed the effect of the number of the electron donor on the thermally activated delayed fluorescent behavior.

## Introduction

Thermally activated delayed fluorescence (TADF), which was firstly reported in 1961 by Parker and Hatchard [1], is a fundamental photophysical phenomenon that refers to delayed fluorescence radiated from the singlet excited state (S_1_) as a consequence of a brief detour to a triplet excited state (T_n_) [i.e., intersystem crossing (ISC) and reverse intersystem crossing (rISC)]. Since the revisit of TADF in organic light-emitting diodes (OLEDs) by Adachi in 2012 [2], TADF-active compounds have emerged as emitters in high-performance organic light-emitting diodes (OLEDs) [3–8], biological probes [9], photocatalysis [10], and some others [11]. Specifically, TADF-active purely organic compounds allow for achieving a very high external quantum efficiency (EQE) of OLEDs without using precious metals such as Ir and Pt in the emitter. Thus, the development of TADF-active organic compounds, the establishment of materials design through systematic structure–property relationship (SPR), and the understanding of the TADF mechanism are highly important tasks in this research field.

The singlet–triplet energy splitting between the S_1_ and T_1_ states (Δ*E*_ST_) and spin–orbit coupling (SOC) play key roles in manifesting the TADF character of an organic compound. To boost the rISC process, ideally, the Δ*E*_ST_ is zero or even negative [12–13], while the SOC is as large as possible. One of the promising molecular design strategies to meet the above-mentioned criteria involves a highly twisted (D)_n_–(A)_m_ (D: electron donor; A: electron acceptor) system, in which efficient intramolecular charge transfer (ICT) occurs in the singlet excited state (^1^CT). An efficient rISC can be mediated by mixing the ^1^CT state with a locally excited triplet state on the donor (^3^LE_D_) or the acceptor (^3^LE_A_) through spin–vibronic coupling [14] or non-Condon effects [15–16].

**Figure 1:**
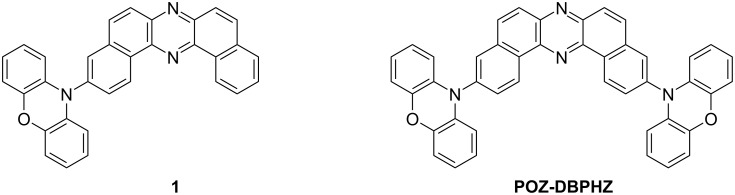
Chemical structures of **1** and **POZ-DBPHZ**.

In 2016, we developed a twisted D–A–D compound **POZ-DBPHZ** (Figure 1) that exhibits efficient orange-to-red TADF [17], and the OLEDs fabricated with **POZ-DBPHZ** achieved a high EQE up to 16%. However, the role of the number of donors and molecular symmetry in the TADF character of **POZ-DBPHZ** remained unexplored, due to the lack of a synthetic method to the asymmetric D–A structure. Herein, we report the synthesis of a new asymmetric D–A compound **1** (Figure 1) as a TADF emitter and its detailed physical properties. Moreover, the developed emitter’s performance was evaluated in an OLED device. To clarify the influence of the donor number and structural symmetry on the physicochemical properties of the DBPHZ-cored D–A system, the properties of D–A compound **1** were compared with those of **POZ-DBPHZ**. Theoretical calculations further support the impact of the donor numbers in the DBPHZ-cored D–A system.

## Results and Discussion

### Synthesis of materials

**Scheme 1:**
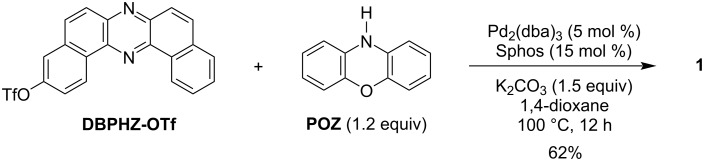
Synthesis of compound **1**.

To synthesize the designed D–A molecule **1**, an asymmetric dibenzophenazine electrophile was required. Recently, we have established a synthetic method for such a compound, i.e., 3-trifluoromethanesulfonyldibenzo[*a,j*]phenazine (**DBPHZ-OTf** in Scheme 1) to prepare linear-type A–D–A–D compounds [18]. Starting from the mono-functionalized compound **DBPHZ-OTf**, the target compound **1** was successfully synthesized through a Pd-catalyzed Buchwald–Hartwig amination with phenoxazine (**POZ**) in a good yield as red-brown solid (Scheme 1). The D–A–D counterpart **POZ-DBPHZ** was synthesized according to the previously reported process [17]. It is noted that the solubility of the D–A compound **1** in organic solvents is lower than that of the D–A–D compound, indicating a more aggregated state of the D–A molecules in the solid state, due to less steric hindrance on the acceptor plane arising from breaking the symmetry. The synthesized compound **1** was fully characterized by ^1^H and ^13^C NMR and IR spectroscopy, MS spectrometry as well as elemental analysis (for the detailed data, see Supporting Information File 1).

### Steady-state PL spectra

**Figure 2:**
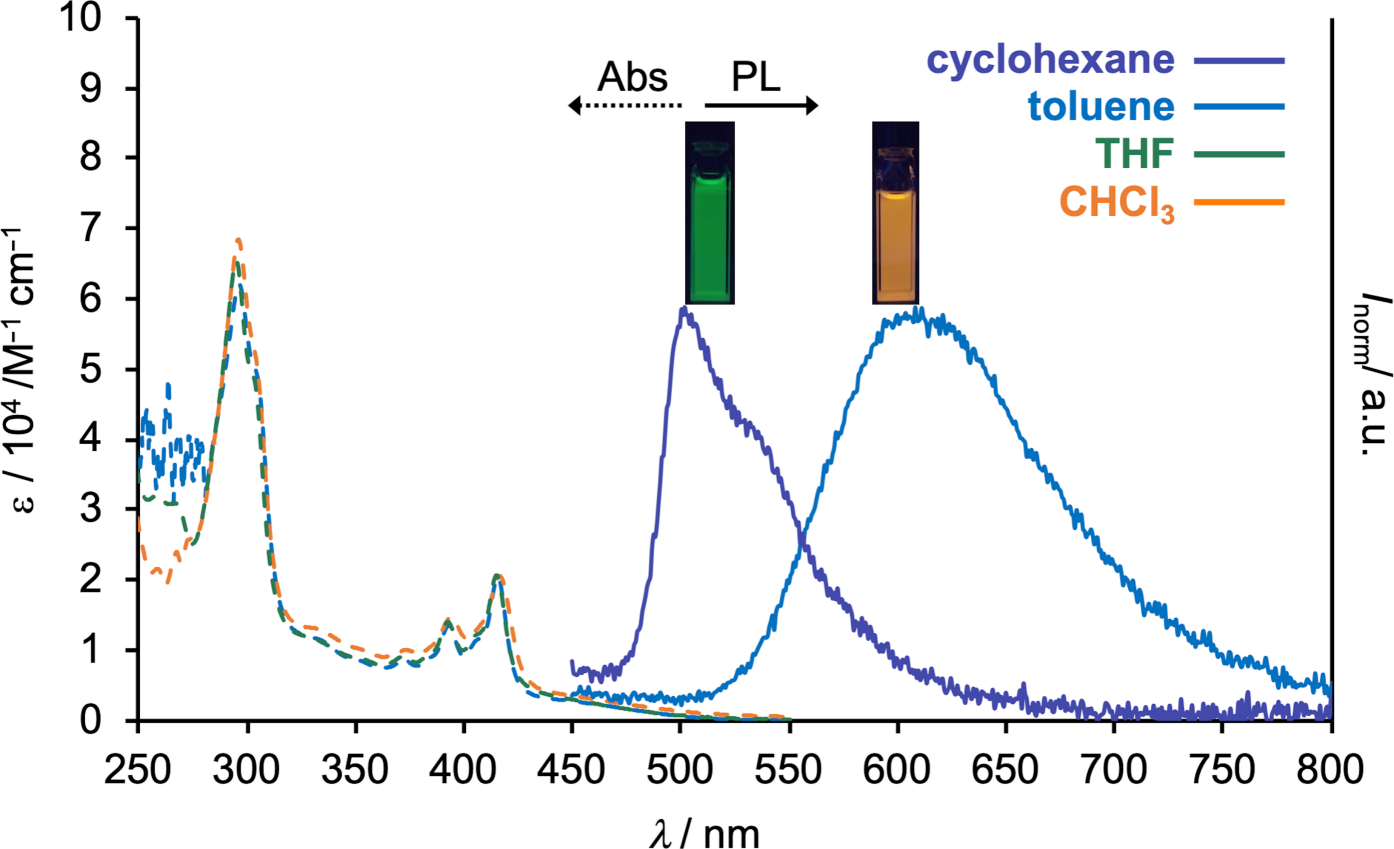
Steady-state UV–vis absorption (Abs) and photoluminescence (PL) spectra of dilute solutions (*c* ≈ 10^−5^ M) of compound **1**. The PL spectra were acquired with λ_ex_ = 340 nm for the cyclohexane solution and λ_ex_ = 360 nm for solutions in the other solvents.

To reveal the photophysical properties of diluted solutions of compound **1**, UV–vis absorption and steady-state photoluminescence (PL) spectra were acquired (Figure 2, and the summary of the properties presented in Table 1). The solutions were prepared with a variety of organic solvents at concentrations of ca. 10^−5^ M. It is noted that the solubility of **1** in cyclohexane is quite low, and thereby the concentration of the cyclohexane solution and the molar absorption coefficient ε were not determined. As is clearly seen from Figure 2, the absorption spectra were not affected by the dielectric constant of the solvents. In contrast, the emission peaks of the PL spectra drastically red-shifted from cyclohexane (λ_PL_ = 502 nm) to toluene (λ_PL_ = 608 nm), and no PL was observed in a more polar solvent such as THF and CHCl_3_ (Figure 2). In addition, the shape of the PL spectrum changed from a vibrationally resolved shape typical of the emission from a locally excited state (^1^LE) to a Gaussian-type broad one typical to the emission from a charge–transfer excited state (CT). The CT emission was totally quenched in a solvent that is more polar than toluene (Figure 2). These photophysical observations are consistent with those of the D–A–D-type compound **POZ-DBPHZ** [17], indicating that one D–A pair is sufficient for generating the CT excited state. In comparison with the photophysical properties of the D–A–D compound, the absorption of **1** (λ_abs_ = 461 nm) is almost the same as that of **POZ-DBPHZ** (λ_abs_ = 463 nm) [17], while the PL emission peak appeared in a slightly blue-shifted region (λ_PL_ = 502 nm) from D–A–D-type compound (λ_PL_ = 521 nm for **POZ-DBPHZ**) in cyclohexane. These data indicate that the effective length of π-conjugation is not affected by the number of donors, probably due to the right D–A dihedral angle for both compounds in the ground state. In contrary, the slight blue-shift of the PL spectra of the D–A compound **1** compared to **POZ-DBPHZ** reflects the contribution of an additional donor to relaxation of the molecular geometry in the excited state. The photoluminescence quantum yield (Φ_PL_) of the D–A compound **1** is lower (0.13 in cyclohexane) than that of the D–A–D compound **POZ-DBPHZ** (0.33) [17], indicating a dominant non-radiative decay of the excited state for the D–A type compound, which was supported by the theoretical calculations (vide infra).

**Table 1:** Summary of steady-state photophysical data of diluted solutions of **1**.^a^

solvent	λ_abs_ (nm)	λ_PL_ (nm)	Φ_PL_^b^

cyclohexane^c^	294, 389, 412	502	0.13
toluene	296, 393, 415	608	0.16
THF	295, 392, 415	–	<0.01
CHCl_3_	295, 394, 416	–	<0.01

^a^Solution concentration: 10^−5^ M; ^b^determined with an integrated sphere; ^c^saturated solution was used, due to the low solubility in cyclohexane.

In the solid state, the D–A compound **1** showed an emission at around λ_em_ = 560 nm with a very low Φ_PL_ (<0.1) (Figure S1 in Supporting Information File 1). The PL spectrum is similar to that in a CBP host matrix (vide infra). The compound **1** showed an aggregation-induced emission (AIE) behavior in a THF/water system, showing a more red-shifted emission peak at around λ_em_ = 600 nm when compared with the as-prepared solid state (Figure S2 in Supporting Information File 1). This indicates that in the as-prepared state and aggregation state the molecular stacking modes should be quite different from each other.

### Time-resolved spectroscopic analysis

**Figure 3:**
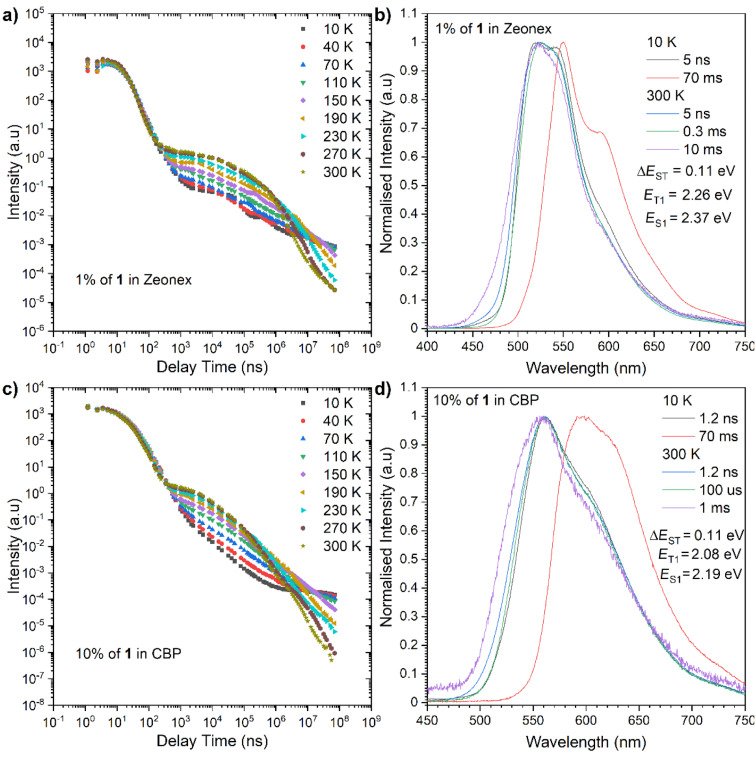
Time-resolved PL decay profiles (intensity vs delay time) and spectra of **1** in a), b) Zeonex^®^ and c, d) CBP matrix. The energies correspond to the maximum emission peaks.

To investigate the delayed fluorescence behavior of the D–A compound **1**, more detailed photophysical studies were performed using a time-resolved spectroscopic technique (Figure 3). Time-resolved photoluminescence (PL) from a blended film (1 wt % of **1** in Zeonex^®^) at 300 K showed two-components emission consisting of a prompt fluorescence (PF) that decays within the order of nanoseconds and a delayed fluorescence (DF) that decays in the range of micro to milliseconds (Figure 3a). These PF and DF spectra are exactly overlapped with each other (Figure 3b), which indicated that both emissions are radiated from the singlet excited state (S_1_). Both emission spectra are not well resolved and in a Gaussian-type shape (Figure 2b), suggesting that these emissions have a mixed character of localized (^1^LE) and charge-transfer state (^1^CT, Figure 3b). The emission from the T_1_ state (phosphorescence, PH) at a low temperature (10 K) with the energy of *E*_T1_ = 2.26 eV showed a similar spectral shape to the phosphorescence spectra of the acceptor core (DBPHZ) [17]. This would indicate that the T_1_ state of the D–A compound is localized on the acceptor unit (^3^LE_A_). The Δ*E*_ST_ of **1** was found to be 0.11 eV, which is twice larger than that of **POZ-DBPHZ** in the same matrix (0.06 eV) [17]. These differences are ascribed to the change in electron density on the acceptor and the electron-donating power of POZ. Therefore, gradual increase of electron-donating strength brings T_1_ energy closer to the acceptor T_1_ energy and leads to a smaller *E*_ST_ gap. But, the activation energy *E*_a_ for the DF process, which was calculated from the Arrhenius plot obtained from the increase of the DF intensity against temperature, was lower for **1** (*E*_a_ = 27 meV) when compared to **POZ-DBPHZ** (*E*_a_ = 47 meV, Table 2) in Zeonex^®^. The directly determined activation energy of the D–A-type compound is half than that of the D–A–D compound, which is in contradiction to the Δ*E*_ST_ value (Table 2). If we support the observation with the DF/PF results that present a stronger TADF property for the mono-substituted derivative **1**, the conclusion of misleading Δ*E*_ST_ comparison can be reached. To avoid confusion, a more effective way is to compare only the activation energy of the DF process.

**Table 2:** Summary of the general photophysical properties of compound **1**.

Compd.	host	λ_em_ [nm]^a^	Φ_PL_^b^	τ_PF_ [ns]^c^	τ_DF_ [μs]^d^	DF/PF^e^	*E*_a_ [eV]^f^	S_1_ [eV]^g^	T_1_ [eV]^g^	*ΔE*_ST_ [eV]^h^

**1**	Zeonex^®^	524	32.5	15.37 ± 1.35	6.9 ± 0.43	5.01	0.028	2.37	2.26	0.11
**POZ-DBPHZ**	Zeonex^®^	530	29.5	10.23 ± 0.16	26.4 ± 1.50	4.72	0.047	2.48	2.40	0.08
**1**	CBP	565	68.5	16.11 ± 0.38	2.96 ± 0.18	0.98	0.015	2.19	2.08	0.11
**POZ-DBPHZ**	CBP	595	79.0	2.7 ± 0.21	0.47 ± 0.04	1.94	0.019	2.28	2.26	0.02

^a^The maximum wavelength of photoluminescence spectra; ^b^photoluminescence quantum yield in degassed; ^c^prompt fluorescence lifetime; ^d^delayed fluorescence lifetime; ^e^the ratio of delayed fluorescence (DF) to prompt fluorescence (PF); ^f^activation energy of the triplet to singlet transfer (error ± 0.01 eV); ^g^singlet and triplet energy (error ± 0.03 eV); ^h^energy splitting (error ± 0.05 eV). All parameters estimated at 300 K.

The time-resolved spectroscopic analysis of the emitter (10 wt % **1**) in an OLED matrix, 4,4’-bis(*N*-carbazolyl)-1,1’-biphenyl (CBP), revealed that the emission at 300 K yields a weaker DF when compared to the Zeonex^®^ matrix (Figure 3c). In addition, the emission in CBP was more complicated, due to the emission spectra that move around with delay time (Figure S3, Supporting Information File 1). At 5.1 ns, the emission peak from PF was observed in a red-shifted region (by approximately 41 nm) than that observed in Zeonex^®^ (λ_em_ = 524 nm) (Figure S3a in Supporting Information File 1). Thereafter, there was a monotonic red shift in the emission peak and the gradual increment during the delay time from 0 ns to 150 ns, and the largest red-shifted spectrum was found at 613 nm (at 150 ns) (Figure S3a, Supporting Information File 1). From 168 ns to 5 μs delay time, the emission peak plateaued at around λ_em_ = 607 nm (Figure S3b, Supporting Information File 1), then from 5 μs to 32 μs, a significant hypsochromic shift of the emission peak was observed down to 560 nm, and the emission peak stayed at this value (Figure S3c, Supporting Information File 1). This behavior brings the proposition that the PF in the nanosecond range based on a CT character with a little contribution from the ^1^LE state have inhomogeneous energies. Firstly, the ^1^LE state decays, and then decays of the lower-energy excited states follow. The triplet energy level of **1** is 2.08 eV, which is lower than that in Zeonex^®^ (2.26 eV). A closer inspection of the transient curves and inset spectra at microsecond delays let us notice that the spectra shift slightly to lower energies (Figure S3 in Supporting Information File 1). This behavior is not unusual in CT-based emitters and can be explained by local interactions between the dipole moment of the host and the excited state dipole moment of the TADF molecule [19].

The activation energy for the TADF process of the D–A compound **1** is as low as 15 meV. Nevertheless, the TADF efficiency of the D–A compound in CBP is much lower when compared to that in Zeonex^®^ and its D–A–D counterpart (Table 2). First, the DF/PF ratio is much smaller in CBP than in Zeonex^®^, suggesting a smaller triplet contribution to the overall emission. If we compare compound **1** with the previously studied D–A–D compound, the Φ_PL_ is slightly lower, but the highest impact is related with DF/PF, where **POZ-DBPHZ** has the twice higher value which in total should give a much lower performance in the device for **1**.

### Thermal stability

To fabricate the OLED devices by thermal evaporation techniques, a high thermal stability is required. To evaluate the effect of the donor number on the thermal stability of the DBPHZ-cored D–A type emitter, the degradation temperature *T*_d_ (5 wt % loss) was investigated by thermogravimetric analysis (TGA), which showed a high *T*_d_ (5 wt % loss under N_2_ atmosphere) of compound **1** (342 °C) (Figure S4a in Supporting Information File 1), which is high enough for a thermal deposition process. However, when compared with the D–A–D counterpart, the *T*_d_ of **1** is much lower (by 111 °C) than that of **POZ-DBPHZ** (453 °C) [17]. These data would support that the increase in the sterically hindered donors in emitting molecule suppress intermolecular contact to enhance the thermal stability.

### OLED fabrication and characterization

**Figure 4:**
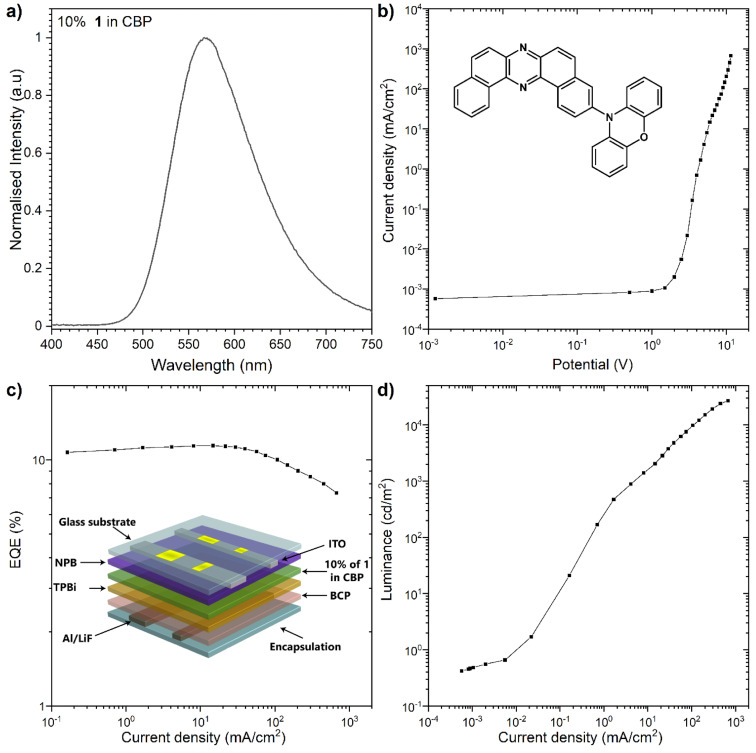
The characteristics of the OLED devices: a) electroluminescence spectra; b) current density-bias characteristics; c) EQE–current density characteristics; d) luminance–current density characteristics.

The OLED device was fabricated and characterized in the CBP host (Figure 4). The HOMO–LUMO values obtained from the electrochemical measurement (Figure S5 in Supporting Information File 1) were used to evaluate whether the emitter works in a previously analyzed device structure [17]. The OLED device structure applied the following configuration: –ITO/NPB [*N*,*N*’-di(1-naphthyl)-*N*,*N*’-diphenyl-(1,1’-biphenyl)-4,4’-diamine] (40 nm)/10% of **1** in CBP (20 nm)/TPBi [2,2’,2’’-(1,3,5- benzinetriyl)-tris(1-phenyl-1*H*-benzimidazole)] (20 nm)/BCP (bathocuproine)] (20 nm)/LiF (1 nm)/Al (100 nm)– (Figure 4). The external quantum efficiency (EQE) was measured at around 11.4%, where the device fabricated with the previously studied D–A–D compound **POZ-DBPHZ** showed 16%. As for luminance, a high luminance of 27,060 cd/m^2^ was obtained, which is slightly lower than that we previously reported (>35,000 cd/m^2^) [17]. A positive aspect about the device fabricated with compound **1** is the lower efficiency roll-off when compared with the previously studied D–A–D emitter. As the result, the efficiency is higher for the D–A compound **1** above 10,000 cd/m^2^, and at the luminance, the EQE was kept around 10%, whereas in the case of doubly donor-substituted compound **POZ-DBPHZ**, the EQE dropped below 10% [17].

### Theoretical calculations

We performed electronic structure calculations on both the D–A (**1**) and D–A–D (**POZ-DBPHZ**) compounds to understand better their respective TADF mechanisms and the efficacy of introducing two electronic donors on the acceptor unit. The calculations employed density functional theory (DFT) with the long-range corrected ωPBE functional and the 6-31G(d,p) basis set. Tuning of the range separation parameter was performed for both molecules [20] with the results collected in Table S1 (Supporting Information File 1). The Tamm–Dancoff (TDA) approximation was used in all excited state calculations and solvent effects were included by means of the polarizable continuum model (PCM) associated with a perturbative state specific solvation method using toluene as solvent. The photophysics of both molecules was analyzed using a unified approach for photophysical rate calculations that employs the nuclear ensemble method as implemented in the NEMO software [21–22] interfaced with the QChem 5.0 program suite [23]. A total of 500 geometries were sampled for each molecule and for each relevant electronic state. From these calculations, emission spectra were computed along with fluorescence, phosphorescence, and ISC rates, providing us insight into the mechanism behind the photophysical behavior of the molecules under analysis in this work.

Taking fluorescence properties as starting point, the simulations point out considerable similarity between the spectra of the D–A and D–A–D compounds. As shown in Figure S6 (Supporting Information File 1), the calculated fluorescence peaks lie at 510 nm and 505 nm for the D–A and D–A–D compounds, respectively. These results agree very well with those obtained from the measurement in Zeonex^®^ (Table 2), which has a similar dielectric constant as toluene (≈2.3), the solvent used in the calculations. A comparison with results from steady-state PL spectra (Table 1), however, show that the predicted peak matches measurements made with cyclohexane, but appear to be blue shifted with respect to measurements in toluene. Considering that cyclohexane has only a slightly lower dielectric constant (≈2.0) than toluene, it is reasonable that calculations would produce similar predictions, which makes the red-shifted experimental emission in toluene more surprising. In addition to similarities in fluorescence energy, the D–A (**1**) and D–A–D (**POZ-DBPHZ**) compounds share very close calculated fluorescence rates (2.2 × 10^7^ s^−1^ and 1.8 × 10^7^ s^−1^, respectively) which translate into prompt fluorescence lifetimes of 45 ns and 54 ns, respectively. These values are in the same order of magnitude as the experimental lifetimes shown in Table 2, further indicating the appropriateness of the theoretical approach.

From the first singlet excited state, we have estimated ISC rates for both molecules. Table S5 in Supporting Information File 1 shows the calculated ISC rates from S_1_ to the first five triplet states. For both molecules, the estimated rate values are comparable or larger than those for fluorescence, which makes the ISC process competitive. Comparing all available processes from the S_1_ state, we are able to estimate probabilities for each transition (detailed in Supporting Information File 1, Table S8 and Table S9). In the case of the D–A compound **1**, the singlet population is expected to split mostly into T_1_ (33%) and T_2_ states (42%), with about 2% probability expected for prompt fluorescence. On the other hand, for the D–A–D compound **POZ-DBPHZ**, transitions to T_1_ display 48% probability whereas fluorescence has around 4%. The remaining probabilities are mostly distributed between transfers to T_2_ and T_3_ with about 20% each.

Transfers to higher lying triplet states may end up relaxing to the lowest triplet state by means of internal conversion. In this sense, it is important to look into the energy gaps between triplet states of both molecules. Considering the average gaps taken from all the conformations sampled in the nuclear ensemble from the T_1_ state geometry, we obtain T_1_ to T_2_ gaps of approximately 0.4 eV for both compounds. This significant value suggests the possibility of the T_2_ population not necessarily decaying to T_1_ instantly. In contrast, the average energy difference between two adjacent triplet levels above T_2_ is approximately 0.1 eV for both molecules, which indicates that internal conversion should be very efficient.

Following the above observations, we estimated rISC rates from the first two triplet states of both molecules and the results are collected in Table S6 and Table S7 (Supporting Information File 1). It is worth noting, that the D–A–D compound **POZ-DBPHZ** presents rISC rates that are larger than those of its D–A counterpart **1** by roughly one order of magnitude, which suggests that the addition of an extra donor unit is able to improve the TADF efficiency. For both triplet states, rISC transfers to S_1_ are overwhelmingly larger than those to higher singlet states. Similarly, these transfer rates to S_1_ are orders of magnitude larger than estimated phosphorescence rates (Table S3 in Supporting Information File 1). The analysis of the probabilities associated with each transfer mechanism from T_1_ and T_2_ (shown in Table S8 and Table S9 of Supporting Information File 1) indicates that the expected depopulation mechanism for the first two triplet states is dominated by an rISC back to the first excited singlet state, which is responsible for the TADF behavior observed in both molecules.

**Figure 5:**
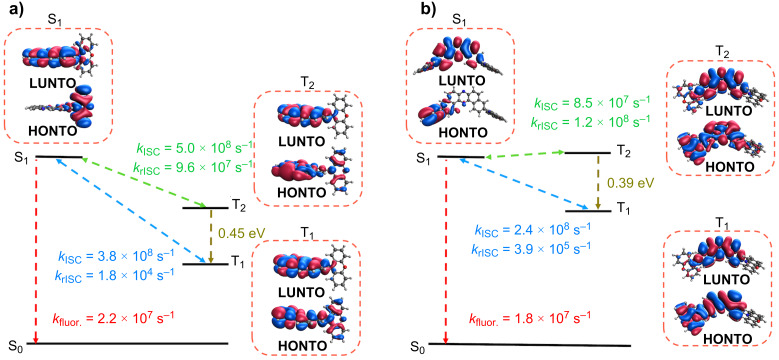
Schematics of the TADF mechanisms along with NTOs for the relevant electronic states for a) D–A compound **1** and b) D–A–D compound **POZ-DBPHZ**.

The rate estimates finally allow us to paint a picture of the TADF mechanism of the two compounds. This is schematically shown in Figure 5, along with the calculated rates for each of the represented processes. In addition, we present natural transition orbitals (NTOs) for the three excited states most relevant for the TADF mechanism. These NTOs demonstrate the similar CT character of the S_1_ state of both compounds, which helps explain their coinciding fluorescence spectra. Finally, the NTOs for the triplet states indicate a possible source for the difference in their TADF efficiencies. Whereas the first two triplet states of the D–A compound **1** correspond mostly to excitations localized in the acceptor fragment, the T_1_ and T_2_ states on the D–A–D molecule **POZ-DBPHZ** display a mixed CT/LE character. It is known that having two states with different electronic characters allows for larger spin–orbit couplings, so we would expect these couplings to be larger in the case of the D–A molecule when comparing with the D–A–D compound. This is indeed the case, as the average spin–orbit coupling for the T_1_ to S_1_ transition in the D–A compound **1** is 0.462 meV, whereas for the D–A–D compound **POZ-DBPHZ** it is 0.177 meV. However, the average energy gap taken from all geometries in the nuclear ensemble for this transition is 0.37 eV for the D–A–D compound and 0.71 eV for the D–A molecule. As such, the higher similarity in electronic character between the singlet and triplet states of the D–A–D molecule was enough to decrease the average energy gap without compromising significantly the spin–orbit coupling, resulting in an overall better TADF performance.

## Conclusion

In conclusion, we have developed a new D–A-type TADF compound and investigated its physicochemical properties for comparison with the corresponding D–A–D analogue. The number of donor units has no effect on the absorption, due to the highly twisted D–A(–D) structures, while an additional donor unit led to a slight red shift in photoluminescence by the stabilization of the charge-transfer singlet excited states (^1^CT). Most importantly, the additional donor unit not only lowers the ^1^CT energy but also is bringing the T_1_ energy to the approximation of the ^3^LE_A_ energy, leading to a narrower singlet–triplet energy gap and a more efficient TADF process, when compared with the mono-donor-substituted compound. On one hand, the comparison of the activation energy for the TADF process for the two compounds gave an inversed order of energy. In addition, the one-less number of donor units in the molecular scaffold led to lower solubility in organic solvents and thermal stability, presumably due to the less steric hindrance around the π-extended conjugated acceptor unit with the unsymmetric molecule structure. The OLEDs fabricated with the D–A emitter achieved a good EQE up to 11%, which exceeds the theoretical maximum (ca. 5%) of prompt fluorescent emitter-based OLEDs. The additional donor gave a better EQE of the OLED device than that fabricated with the D–A compound, due to a less efficient TADF process. Taken together the experimental and theoretical calculations, the role of the additional donor unit in the TADF mechanism is boosting the rISC process by balancing the singlet–triplet energy gap and spin–orbit coupling. The results showcased herein would allow for designing efficient TADF emitters more flexibly in the future.

## Supporting Information

File 1 General information, synthetic procedures, spectral data, photophysical data, and theoretical calculation data.
