# Supplementary material for: Comparative study of thermally activated delayed fluorescent properties of donor–acceptor and donor–acceptor–donor architectures based on phenoxazine and dibenzo[a,j]phenazine
Source: Beilstein J Org Chem. 2022 Apr 25;18:459–68. doi: 10.3762/bjoc.18.48 (PMC9062652; doi:10.3762/bjoc.18.48)
Supplement: File 1 — General information, synthetic procedures, spectral data, photophysical data, and theoretical calculation data. [file Beilstein_J_Org_Chem-18-459-s001.pdf]

BEILSTEIN JOURNAL OF ORGANIC CHEMISTRY

Supporting Information

for

Comparative study of thermally activated delayed fluorescent properties of donor–acceptor and donor–acceptor–donor architectures based on phenoxazine and dibenzo[*a,j*]phenazine

Saika Izumi, Prasannamani Govindharaj, Anna Drewniak, Paola Zimmermann Crocomo, Satoshi Minakata, Leonardo Evaristo de Sousa, Piotr de Silva, Przemyslaw Data and Youhei Takeda

Beilstein J. Org. Chem. **2022,** *18,* 459–468. doi:10.3762/bjoc.18.48

General information, synthetic procedures, spectral data, photophysical data, and theoretical calculation data

License and Terms: This is a supporting information file under the terms of the Creative Commons Attribution License (https://creativecommons.org/licenses/by/4.0). Please note that the reuse, redistribution and reproduction in particular requires that the author(s) and source are credited and that individual graphics may be subject to special legal provisions.
The license is subject to the *Beilstein Journal of Organic Chemistry* terms and conditions: (https://www.beilstein-journals.org/bjoc/terms)

Table of contents

General remarks S1

Synthetic procedures and spectroscopic data of new compounds S2

Additional spectroscopic analysis S3

Thermogravimetric analysis S4

Electrochemical analysis S4

Copies of NMR spectra S5

Theoretical calculations S6–S9

References S10

General remarks. All reactions were carried out under an atmosphere of nitrogen unless otherwise noted. Products were purified by chromatography on silica gel Chromatorex NH (Fuji Silysia Chemical Ltd.). Analytical thin-layer chromatography (TLC) was performed on pre-coated silica gel glass plates (Fuji Silysia Chromatorex NH, 0.25 mm thickness). Compounds were visualized under UV light. Melting points were determined on a Stanford Research Systems MPA100 OptiMelt Automated Melting Point System or a DSC 6220 (SII) system. All ^{1}H and ^{13}C NMR spectra were recorded on a JEOL JMTC-400/54/SS Spectrometer (^{1}H NMR, 400 MHz; ^{13}C NMR, 100 MHz) using tetramethylsilane as an internal standard. Infrared spectra were acquired on a SHIMADZU IRAffinity-1 FT-IR Spectrometer. Mass spectra and high-resolution mass spectra were obtained on a JEOL JMS-700 Mass Spectrometer. The elemental analysis (CHN) was carried out with a JM10 (J-SCIENCE LAB CO., Ltd). UV–vis spectra were recorded on a Shimadzu UV-2550 spectrophotometer. Steady-state emission spectra were recorded on a HAMAMATSU C11347-01 spectrometer with an integrating sphere. Thermogravimetric analysis (TGA) was performed with TG/DTA-7200 system (SII Nano Technology Inc.).

Materials. Dehydrated 1,4-dioxane was used as received. Dibenzo[*a*,*j*]phenazin-3-yl trifluoromethanesulfonate [CAS No. 2409482-67-1][S1] was synthesized according to the reported procedure. Solvents of fluorescence spectroscopic grade for measurement of UV–vis and emission spectra were purchased from Nacalai Tesque Inc.

Steady-state UV–vis absorption and PL spectra. All the steady-state UV–vis absorption and PL spectra were measured at room temperature with diluted solutions (10^{-5} M) except for saturated cyclohexane solution (c is less than 10^{-6} M, due to the poor solubility of the material in cyclohexane), which were prepared from degassed spectroscopic grade solvents (N_2 for 30 min).

Synthetic procedures and spectroscopic data of new compounds.

Synthesis of 10-(dibenzo[a,j]phenazin-3-yl)-10H-phenoxazine (1).

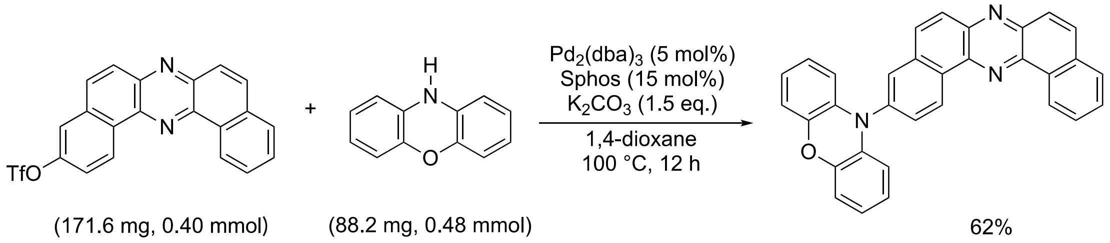

Dehydrated 1,4-dioxane was degassed through freeze-pump-thaw cycling for 3 times before use. A two-necked reaction tube (10 mL) equipped with a magnetic stirring bar, a rubber septum, and a glass stopper was flame-dried under reduced pressure and then purged with N_2 gas for 3 times. To the tube, were added dibenzo[*a*,*j*]phenazin-3-yl trifluoromethanesulfonate (171.6 mg, 0.40 mmol), phenoxazine (88.2 mg, 0.48 mmol, 2.2 equiv), $Pd_2(dba)_3$ (18.3 mg, 20 μmol, 5.0 mol %), SPhos (24.8 mg, 60 μmol, 15 mol %), K_2CO_3 (83.4 mg, 0.60 mmol, 1.5 equiv), and 1,4-dioxane (3 mL) under a stream of N_2 gas, and the mixture was stirred on an aluminum heating block set at 100 °C for 12 hours. After the mixture was allowed to cool to room temperature, water (5 mL) was added to the reaction mixture, which was extracted with $CHCl_3$ (20 mL × 3). The combined organic extracts were dried over Na_2SO_4, and the solvent was removed under reduced pressure to give the crude product. The crude product was purified by flash column chromatography on NH silica gel (eluent: *n*-hexane/EtOAc 10:0–8:2, then $CHCl_3$). The resulting solid was reprecipitated from $CHCl_3$/MeOH to give the title compound as red-brown solid in 62% yield (114.3 mg, 0.25 mmol). mp 331 °C (dec.); R_f 0.40 (*n*-hexane/EtOAc 8:2, NH silica); ^{1}H NMR (400 MHz, $CDCl_3$) δ 9.86 (d, J = 8.4 Hz, 1H), 9.65 (d, J = 7.2 Hz, 1H), 8.18–8.10 (m, 4H), 8.02–8.01 (m, 2H), 7.89–7.82 (m, 3H), 6.76 (dd, J = 7.6, 1.2 Hz, 2H), 6.69 (ddd, J = 7.6, 7.6, 1.2 Hz, 2H), 6.56 (ddd, J = 7.6, 7.6, 1.2 Hz, 2H), 6.03 (dd, J = 7.6, 1.2 Hz, 2H); ^{13}C NMR (100 MHz, $CDCl_3$) δ 144.0, 143.2, 142.9, 140.9, 140.0, 139.8, 135.3, 134.3, 133.4, 132.9, 131.6, 131.2, 131.1, 130.2, 129.9, 129.6, 128.5, 128.2, 128.0, 127.9, 126.9, 125.2, 124.4, 121.6, 115.6, 113.4; IR (ATR, cm^{-1}): 3047, 1593, 1487, 1332, 1292, 1271, 846, 796, 746, 723, 700; MS (FAB$^+$): *m/z* (relative intensity, %): 462 ([M+1]$^+$, 37), 461 (M$^+$, 100), 271 ([$C_{12}H_8NO$]$^+$, 32); HRMS (FAB$^+$): *m/z* calcd for $C_{32}H_{19}N_3O$ (M) 461.1528, found 461.1525; Anal. Calcd for $C_{32}H_{19}N_3O$: C, 83.28; H, 4.15; N, 9.10. found: C, 83.08; H, 3.91; N, 8.91.

Additional spectroscopic analysis.

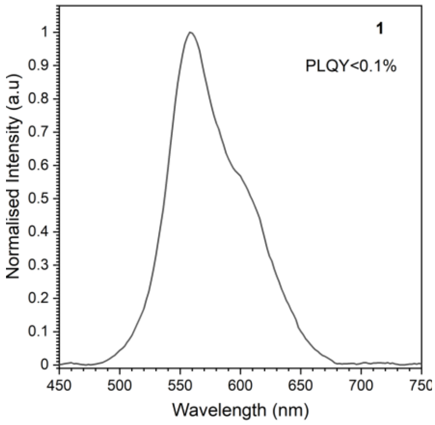

Figure S1: Solid-state PL spectrum of **1**.

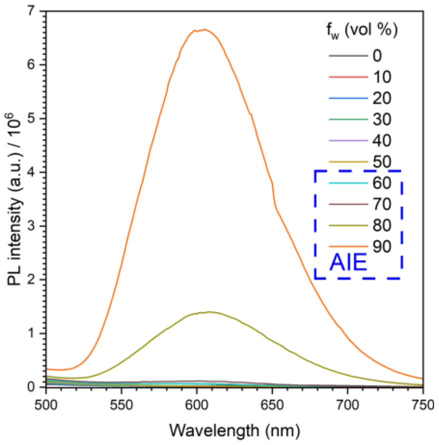

Figure S2: Emission spectra (λ_{ex} = 355 nm) of 100 µM **1** in different THF/water ratio blends.

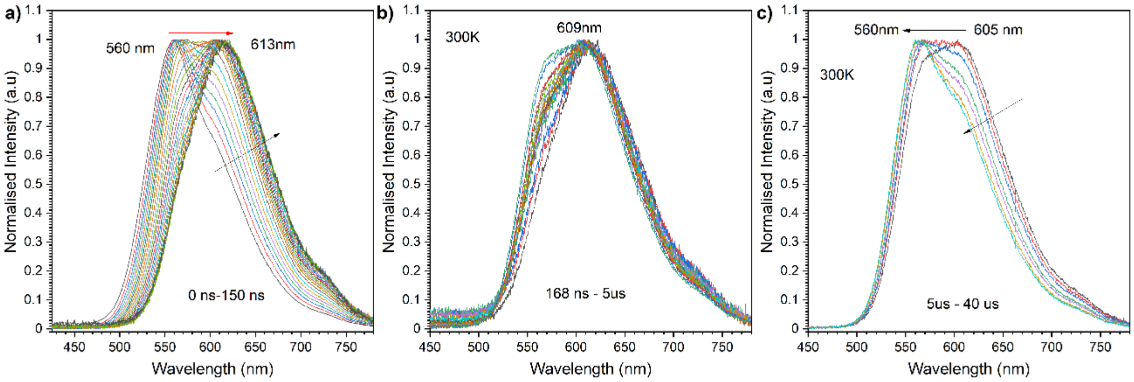

Figure S3: Time-resolved photoluminescence (PL) spectra of **1** in CBP with the delayed time.

Thermogravimetric analysis (TGA).

The TGA profiles of **1** were acquired using a Pt pan under air or N_2 gas flow (200 mL/min), starting from 40 °C to 1000 °C at a ramp rate of 10 °C/min.

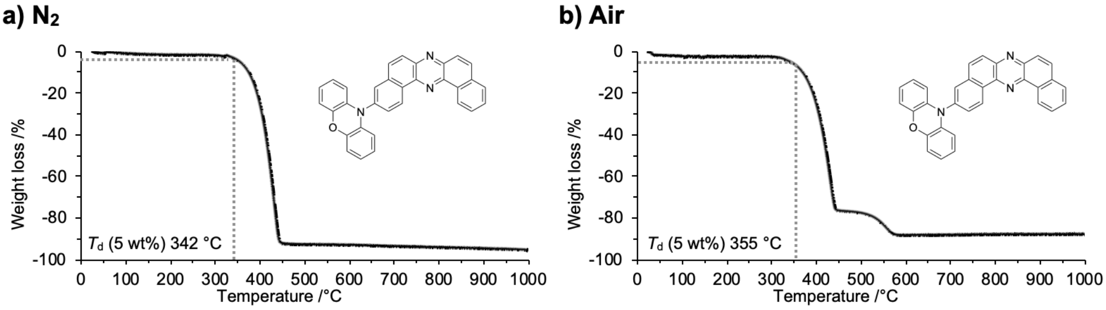

Figure S4: TGA profiles of compound **1** under a) N_2 gas flow and b) air.

Electrochemical analysis.

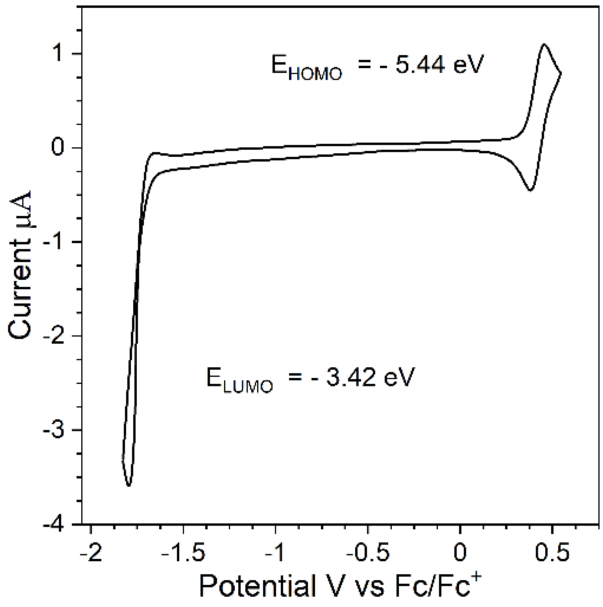

Figure S5: CV of 1 mM of **1** in 0.1 M Bu_4NBF_4 in DCM electrolyte at a scan rate of 50 mV/s.

Copies of NMR spectra.

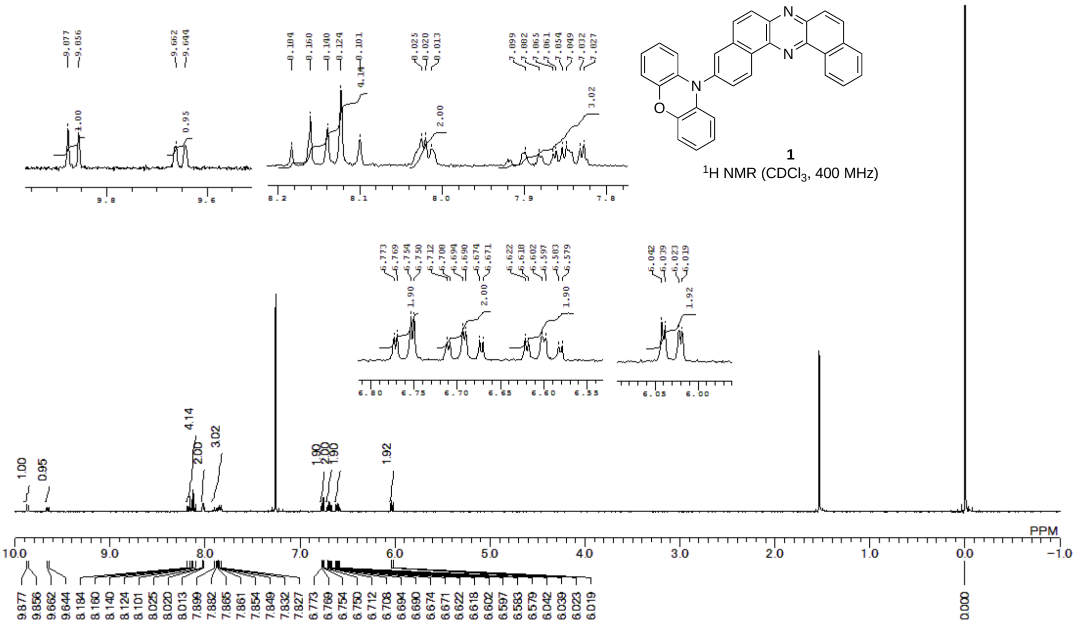

1

^{1}H NMR ($CDCl_3$, 400 MHz)

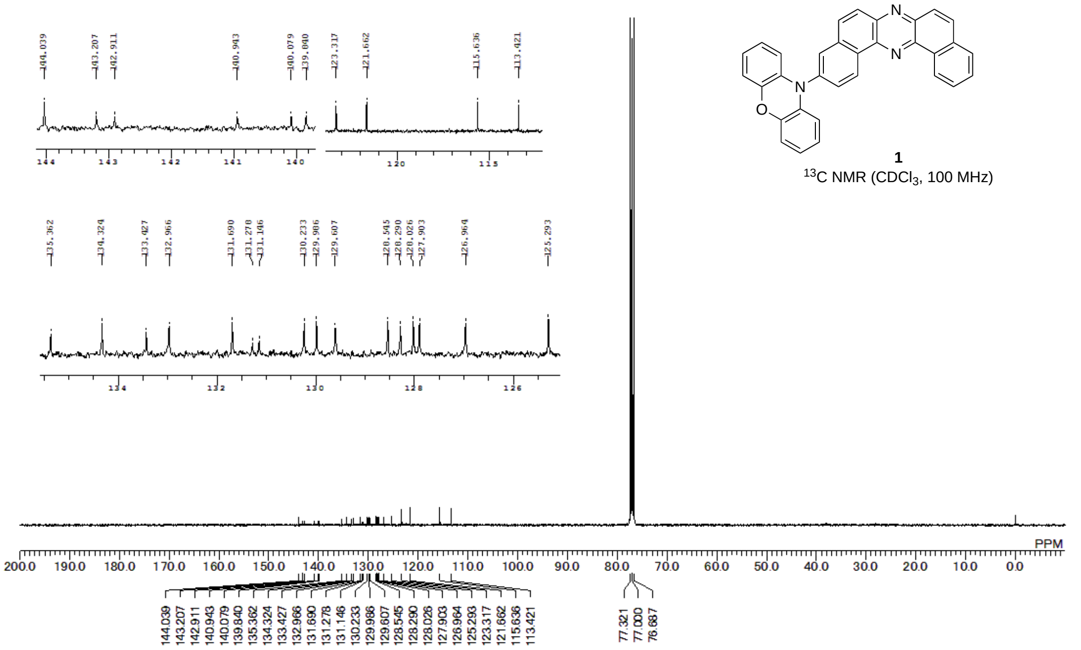

1

^{13}C NMR ($CDCl_3$, 100 MHz)

Theoretical calculations.

Fluorescence, phosphorescence, and ISC rates were estimated with the nuclear ensemble method, as implemented in the NEMO software interfaced with QChem 5.0. A total of 500 geometries were sampled taking as starting point the optimized geometries of the DA and DAD compounds at either S_1, T_1, or T_2 states.

Functional tuning

Table S1: Tuned values of the long-range separation parameter for the different compounds.

Compound	ω ($bohr^{-1}$)
**1**	0.191
**POZ-DBPHZ**	0.181

Fluorescence simulations

Table S2: Calculated fluorescence rates in toluene for the 2 compounds in their preferred conformation in the S_1 state.

Compound	Rate (s^{-1})	Error (s^{-1})
**1**	2.2E+07	0.6E+07
**POZ-DBPHZ**	1.8E+07	0.6E+07

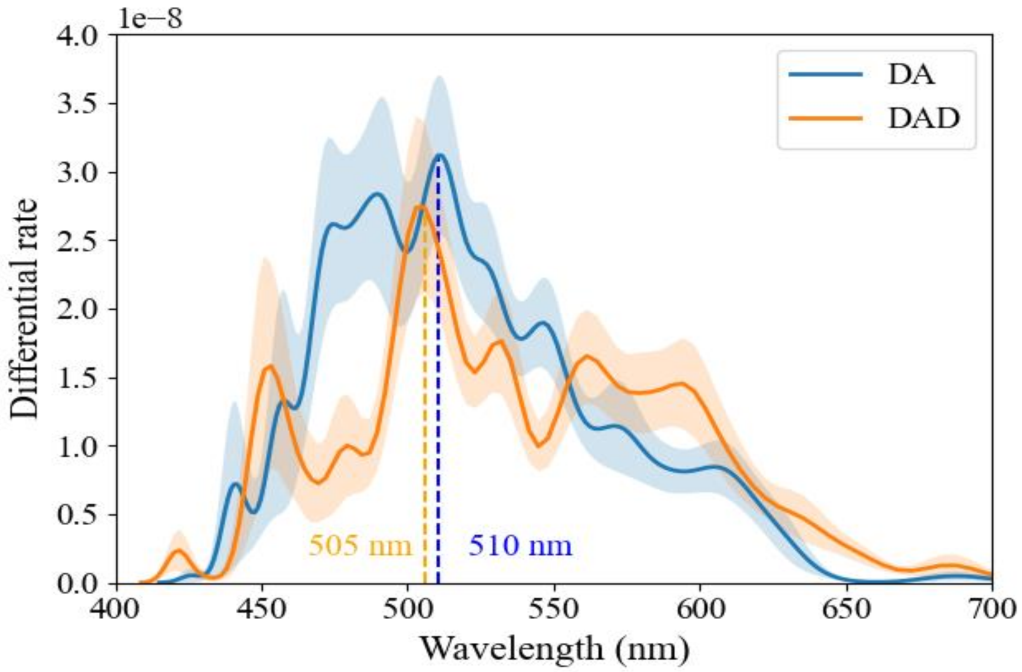

Figure S6: Simulated fluorescence spectra for the two compounds. The shadowed region marks the calculated uncertainty.

Phosphorescence simulations

Table S3: Calculated T_1 and T_2 phosphorescence rates in toluene for the 2 compounds in their preferred conformation in the T_1 state.

Compound/State	T_1		T_2	
	Rate (s^{-1})	Error (s^{-1})	Rate (s^{-1})	Error (s^{-1})
**1**	9.1E+00	6.6E+00	2.0E+04	2.6E+04
**POZ-DBPHZ**	3.7E+01	6.5E+01	9E+04	16E+04

Intersystem crossing rates

Table S4: Calculated reorganization energies used in the ISC calculations for singlet-to-triplet and triplet-to-singlet transitions.

Compound	S -> T (eV)	T -> S (eV)
**1**	0.281	0.366
**POZ-DBPHZ**	0.022	0.359

Table S5: Estimated ISC rates from the S_1 state for the 2 compounds.

ISC	**1**		**POZ-DBPHZ**	
Transfer	Rate (s^{-1})	Error (s^{-1})	Rate (s^{-1})	Error (s^{-1})
S_1->T_1	3.8E+08	0.5E+08	2.4E+08	1.2E+08
S_1->T_2	5.0E+08	1.8E+08	8.5E+07	2.5E+07
S_1->T_3	2.1E+08	1.2E+08	9.5E+07	4.9E+07
S_1->T_4	4.9E+07	2.5E+07	4.8E+07	3.5E+07
S_1->T_5	7.8E+06	4.4E+06	8.6E+06	3.4E+06

Table S6: Estimated ISC rates from the T_1 state for the 2 compounds.

ISC	**1**		**POZ-DBPHZ**	
Transfer	Rate (s^{-1})	Error (s^{-1})	Rate (s^{-1})	Error (s^{-1})
T_1->S_1	1.8E+04	1.0E+04	3.9E+05	1.3E+05
T_1->S_2	7.1E+01	6.8E+01	2.5E+04	0.8E+04
T_1->S_3	2.3E-02	2.3E-02	3.5E+03	3.4E+03
T_1->S_4	8.3E-10	3.9E-10	4.6E-01	4.0E-01
T_1->S_5	1.5E-14	0.7E-14	2.4E-05	1.4E-05

Table S7: Estimated ISC rates from the T_2 state for the 2 compounds.

ISC	**1**		**POZ-DBPHZ**	
Transfer	Rate (s^{-1})	Error (s^{-1})	Rate (s^{-1})	Error (s^{-1})
T_2->S_1	9.6E+07	1.8E+07	1.2E+08	0.09E+08
T_2->S_2	1.7E+04	1.2E+04	1.5E+07	0.2E+07
T_2->S_3	1.3E+02	1.1E+02	5.1E+04	2.6E+04
T_2->S_4	9.6E-02	5.1E-02	1.5E+02	0.6E+02
T_2->S_5	2.3E-05	1.7E-05	1.3E+00	0.6E+00

Table S8: Estimated probabilities for transfers from the S_1, T_1, and T_2 states for compound **1**.

Transfer	Probability (%)	Transfer	Probability (%)	Transfer	Probability (%)
S_1->T_1	32.7	T_1->S_1	99.5	T_2->S_1	100.0
S_1->T_2	42.3	T_1->S_2	0.4	T_2->S_2	0.0
S_1->T_3	18.2	T_1->S_3	0.0	T_2->S_3	0.0
S_1->T_4	4.2	T_1->S_4	0.0	T_2->S_4	0.0
S_1->T_5	0.7	T_1->S_5	0.0	T_2->S_5	0.0
S_1->S_0	1.9	T_1->S_0	0.1	T_2->S_0	0.0

Table S9: Estimated probabilities for transfers from the S_1, T_1, and T_2 states for compound **POZ-DBPHZ**.

Transfer	Probability (%)	Transfer	Probability (%)	Transfer	Probability (%)
S_1->T_1	48.2	T_1->S_1	93.1	T_2->S_1	88.4
S_1->T_2	17.2	T_1->S_2	6.1	T_2->S_2	11.5
S_1->T_3	19.4	T_1->S_3	0.8	T_2->S_3	0.0
S_1->T_4	9.7	T_1->S_4	0.0	T_2->S_4	0.0
S_1->T_5	1.7	T_1->S_5	0.0	T_2->S_5	0.0
S_1->S_0	3.7	T_1->S_0	0.0	T_2->S_0	0.1

References

S1. Izumi, S.; Higginbotham, H. F.; Nyga, A.; Stachelek, P.; Tohnai, N.; Silva, P. de; Data, P.; Takeda, Y.; Minakata, S. *J. Am. Chem. Soc.* **2020**, *142* (3), 1482–1491.
